# Risk factors for postoperative seizures in patients with chronic subdural haematomas

**DOI:** 10.1007/s10143-022-01858-5

**Published:** 2022-09-12

**Authors:** Andreas Kramer, Xenia Degenhartt, Angelika Gutenberg, Florian Ringel

**Affiliations:** 1 Department of Neurosurgery, University Medical Centre Mainz, Johannes Gutenberg University, Mainz, Germany; 2 Department of Neurosurgery, Asklepios Klinikum Hamburg-Harburg, Hamburg, Germany

**Keywords:** Chronic subdural haematoma, Burr hole trepanation, Haematoma evacuation, Postoperative seizures, Subdural drain, Haematoma membranes

## Abstract

Postoperative seizures are a frequently occurring yet not well-understood complication in patients undergoing surgical treatment of chronic subdural haematomas (cSDHs). Therefore, we investigated surgical and non-surgical risk factors that are commonly considered causal in provoking epileptic seizures, paying special attention to the intracranial course of the subdural drain (SDD) and the configuration of the haematoma. Data of patients with a cSDH, that were treated at our neurosurgical department between 2008 and 2014 were analysed. Patients suffering from severe pre-existing conditions and those who have been treated conservatively were excluded. Epidemiologic data as well as relevant clinical data were collected. Pre- and postoperative CT scans were analysed regarding morpho- and volumetric parameters. In order to objectify the influence of the SDD, its intracranial course and localisation (entering angle as well as the angle between drain and brain surface) were measured. For statistical analysis, univariate and multiple logistic regression models as well as Fisher’s exact test were used. Two hundred eleven consecutive patients have been included. Mean age was 75.6 years, and 69% were male. Nineteen (9%) patients suffered from postsurgical seizures. Membranes within the haematoma were present in 81.5%. Pre- to postoperative haematoma reduction was significant (mean of difference − 12.76 mm/ − 9.47 mm in coronal/axial CT planes, *p* = 0.001/ < 0.001). In 77.9%, SDD showed cortical contact with eloquent regions and had an unfavourable course in 30 cases (14.2%). Surgical complications consisted of cortical bleeding in 2.5%, fresh subdural haematoma in 33.5% and wound infections in 1.4% of patients. Neither in univariate nor in multiple regression analyses any of the following independent variates was significantly correlated with postsurgical seizures: pre-existing epilepsy, alcohol abuse, right-sided haematomas, localization and thickness of haematoma, presence of septations, SDD-localization and to-brain angle, subdural air, and electrolyte levels. Instead, in multiple regression analyses, we found the risk of postsurgical seizures to be significantly correlated and increased with left-sided cSDH treated via craniotomy (*p* = 0.03) and an unfavourable course of the SDD in left-sided cSDH (*p* = 0.033). Burr hole trepanation should be preferred over craniotomy and care must be taken when placing a SDD to avoid irritating cortical tissue. The configuration of the haematoma does not appear to affect the postoperative seizure rate.

## Introduction

Chronic subdural haematomas arise commonly as a result of a minor head trauma and are one of the most frequently treated diseases in neurosurgical practice. Due to natural brain atrophy, accompanying elongation of subdural bridging veins and necessity of taking anticoagulants the incidence of cSDHs increases with age. Clinical presentation is often nonspecific with symptoms ranging from headache to vigilance impairment, focal neurological deficits or hemiparesis. A cranial computed tomography (CCT) represents the diagnostic medium of choice allowing a valid assessment of the haematoma configuration and age with commonly broad and fast availability. Surgical treatment options of symptomatic cSDH comprise bed-sided twist drill craniotomy, burr hole trepanation and mini-craniotomy with or without insertion of a subdural or subgaleal drain.

Focal, generalised or secondary generalised epileptic seizures represent a postoperative complication, that can negatively affect postoperative recovery [1–3] or even increase mortality rate [4, 5]. The reported incidence of postoperative seizures varies between 2.3% [6] and 17% [7]. Generally considered risk factors are pre-existing epilepsy, alcohol withdrawal in case of underlying dependency or electrolyte imbalance as well as the presence of granulation membranes within the haematoma cavity [8]. There has been a variety of studies investigating the benefit of perioperative administration of antiepileptic drugs (AED), resulting in heterogenous and partially contrary recommendations on the use of AED [5, 6, 9]. The lack of a reliable evidence for occurrence and avoidance of postoperative seizures underlines the importance of a profound understanding of risk factors so that they can be taken into consideration by the treating surgeon. The objective of the present study is to evaluate risk factors for postoperative seizures that are modifiable by the treating surgeon paying special attention to the anatomical localisation of the SDD and the technique of haematoma removal.

## Material and methods

In a retrospective analysis data of all patients who presented with a newly diagnosed or recurrent cSDH to our department between 2008 and 2014 were reviewed. Patients were included in the analysis if they (i) had a cSDH, (ii) underwent surgical haematoma evacuation, and (iii) had imaging studies available for analysis. Patients were excluded (i) if conservative treatment was chosen and (ii) upon relevant comorbidities, such as severe traumatic brain injury leading to the development of a cSDH.

**Fig. 1:**
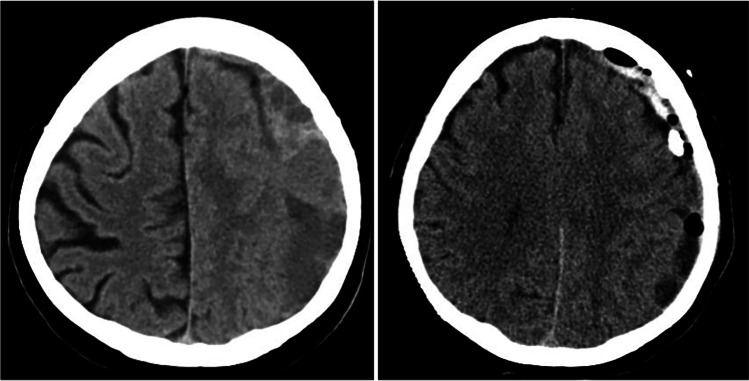
Case illustration of a 75-year-old female patient presenting with dysphasia. The preoperative CCT scan (left) shows a space-occupying multi-layered cSDH of the left hemisphere. In view of the heterogeneous configuration of the haematoma left-sided mini craniotomy was chosen as a surgical approach. After an initially regular postoperative course with a resolution of any symptoms, she developed aphasia. The CCT scan performed immediately revealed intracranial air entrapment and fresh blood next to the craniotomy and the SDD. The SDD was removed and anticonvulsive therapy initiated. No further seizures occurred

According to our departmental clinical routine patients with a symptomatic cSDH or a relevantly sized haematoma underwent burr hole trepanation or mini-craniotomy for haematoma evacuation. In general, burr hole trepanation was the preferred surgical approach, also in cases with partial acute bleeding and/or septations within the haematoma. However, it was up to the treating surgeon to perform a mini craniotomy, e.g. when relevant amounts of acute bleeding or multi-layer septations were present. Figure 1 illustrates a case in which mini-craniotomy was chosen as a surgical approach. In all cases, sterile saline solution at body temperature was used to flush out the haematoma and a subdural drain (SDD) was inserted for further haematoma drainage. The drain was removed after suspension of haematoma drainage and adequate CCT imaging results, routinely acquired within 2 days after surgery. During the study period the departmental imaging algorithm of operatively treated patients with cSDH comprised CCT scans pre- and postoperatively prior to and after removal of the SDD.

Relevant data on patients’ status was collected including demographic characteristics, pre-existing epilepsy, alcohol abuse, epileptogenic medication (antipsychotics, antidepressants), occurrence of postoperative seizures, type of treatment of postoperative seizures (removal of SDD/administration of anticonvulsive drugs), postoperative complications, potential alcohol withdrawal, serum sodium, potassium, calcium, chloride and glucose concentration. Furthermore, the surgical approach was analysed.

**Fig. 2:**
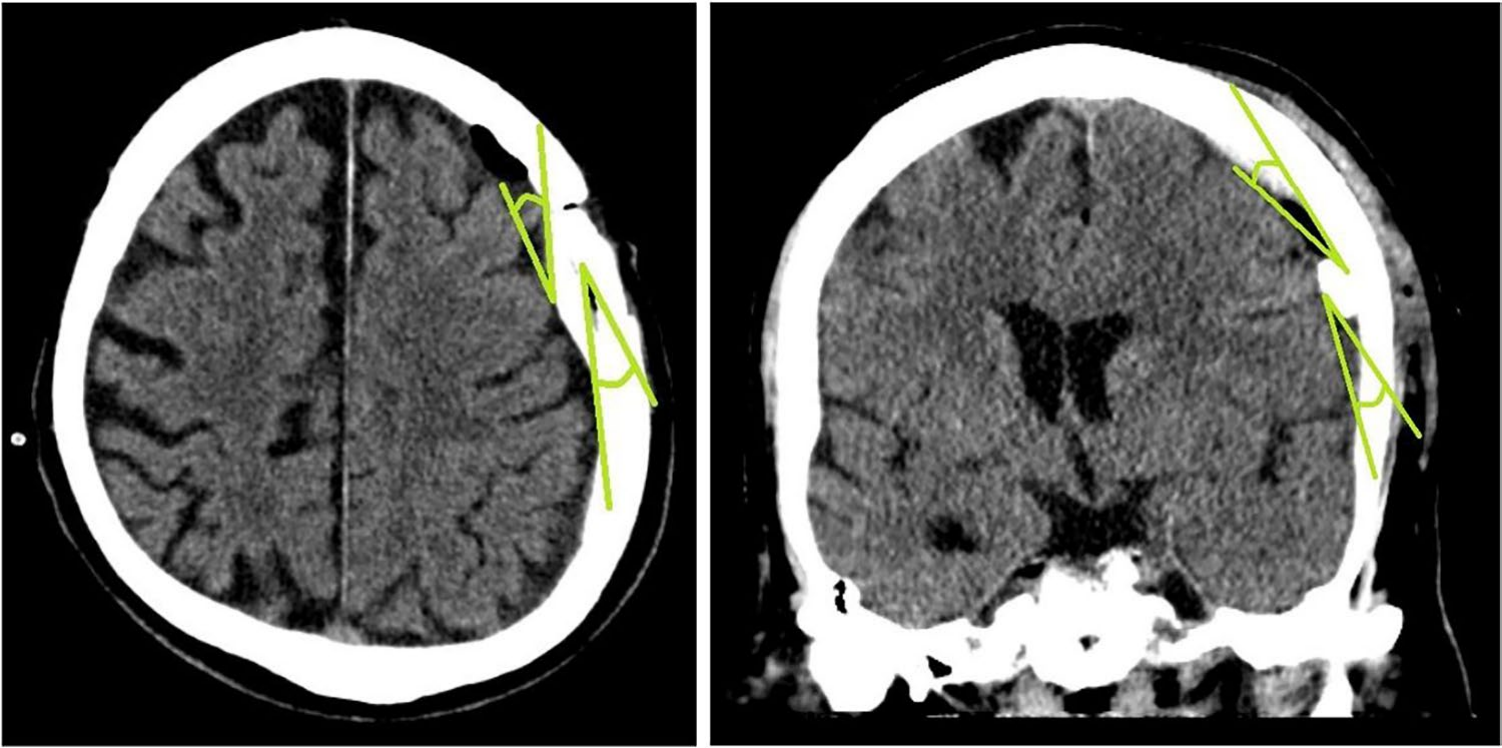
Entry angle of the SDD in axial and coronal planes in degrees, measured as the angle between the inner side of the calvaria at the level of the burr hole and the first segment of the drain after entering the subdural space and between the tangent of the cortical surface and the first segment of the drain

**Fig. 3:**
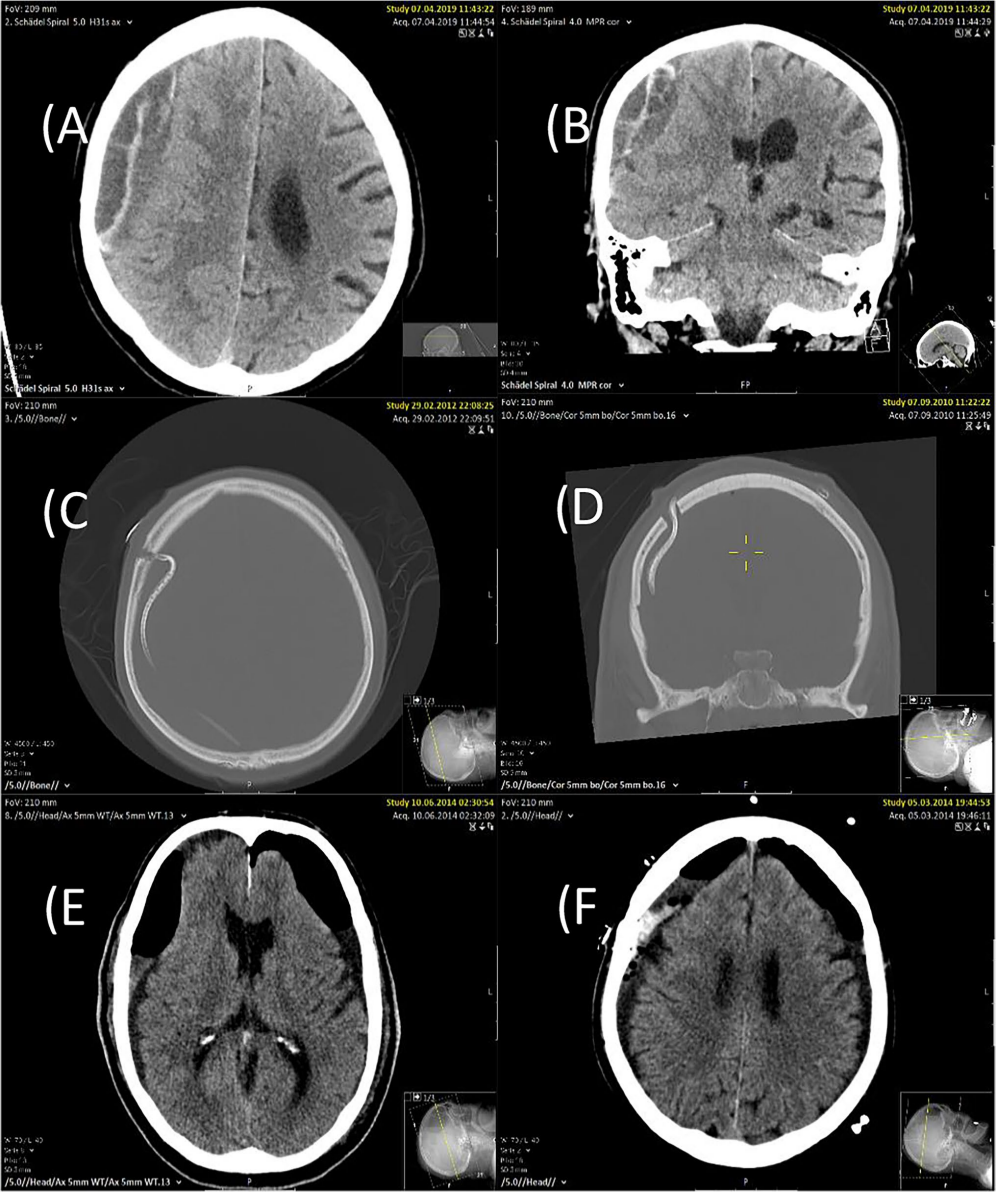
**A**,** B** Septations within the haematoma. **C** A case of misplacement and consecutively unfavourable course of the SDD with an excessively and unusual long intracranial course. **D** Unfavourable (curled) intracranial course of the SDD. **E** Space-occupying air entrapment. **F** Fresh blood clot postoperatively

Pre- and postoperative CCT and MRI scans were evaluated using the ConVis DICOM Viewer 2004 (Version 2.8.14, ConVis Med. DV GmbH & Co. KG). The following radiographic characteristics were assessed in pre- and postoperative imaging: Localisation of the haematoma, density of the haematoma, pre- and postoperative maximum axial and coronal extent of the haematoma, midline shift (distance of the midline to the foramen of Monro in axial plane), presence of membranes within the haematoma. Postoperative CCT scans were assessed for the axial and coronal entering angle (Fig. 2) as well as the intracranial course of the SDD, the angle between the distal end of the drain and the cortex surface as well as the postoperative intracranial air volume. The course of the SDD was termed unfavourable in those cases where it did not maintain a straight line but a meandered or curled course in postoperative CCT scans (Fig. 3).

Statistical analysis was performed using IBM SPSS (IBM, USA). Statistical significance was accepted at an error probability of *p* ≤ 0.05. Univariate and multiple logistic regression models as well as Fisher’s exact test were used. Regression models were applied separately for left- and right-sided haematomas and for the total of all haematomas. In covariate selection for the multiple logistic regression model, multicollinearity issues were avoided by assessing pairwise associations between potential covariates. Covariates with strong pairwise associations were not considered in the model because of difficulties in interpreting their parameter estimates.

## Results

### Patient baseline characteristics

A total of 211 patients were treated for 248 primary diagnosed and 40 residual or recurrent cSDHs. One hundred forty-five men (69%) and 66 women (31%) with a mean age of 76 years (27–93 years) were assessed. Women were significantly older than men at the time of treatment (78 vs. 74 years, *p* = 0.011). Twelve patients (5.69%) were known to be suffering from epilepsy. Four patients (1.9%) were addicted to alcohol and 17 patients (6.7%) had a history of cranial surgery that did not relate to the occurrence of the subdural haematoma.

Average in hospital stay of first-time treated patients was 11.67 days ranging from 2 to 56 days (median 10 days). When a second surgery was necessary due to early recurrence or haematoma residuals average in hospital stay was 14.1 days ranging from 4 to 78 days (median 9 days).

### Haematoma characteristics

A majority of 98 haematomas (46.2%) was located on the left side, 76 haematomas (35.8%) were located on the right side and 37 (17.5%) were bilateral. Most haematomas were localised over the frontoparietal (38.3%) or frontoparietotemporal (36.3%) cortex. In the majority of cases radiographic signs of membranes within the haematoma were present (*n* = 202, 81.5%). 148 (59.67%) haematomas showed an inhomogeneous density in preoperative imaging.

Concerning the evaluation of the pre- to postoperative haematoma ratio, the mean preoperative thickness of all haematomas was 20.34 mm in the axial plane (6–64 mm) and 21.55 mm in the coronal plane (8–66 mm). Postoperatively, the mean haematoma thickness was significantly reduced to 10.87 mm in axial planes, ranging from 0 to 30 mm (Ø -9.47 mm/-46.56%; 95% CI 8.67–10.27; *p* < 0.001) and to 9.42 mm in coronal planes, ranging from 0 to 31 mm (Ø − 12,76 mm/ − 59.21%; 95% CI 11.43–14.1; *p* = 0,001).

Average midline shift in the axial plane was 6.41 mm pre- and 3.54 mm postoperatively (− 2.87 mm/ − 44.77%; 95% CI 2.35–3.38; *p* < 0.001).

### Surgical approach

In 182 patients (73.4%), a single burr hole was used as a surgical approach, 38 patients (15.3%) were treated via osteoplastic mini-craniotomy and in 24 cases (9.7%) a twist-drill burr hole in local anaesthesia was performed. Three haematomas (1.2%) were evacuated using a frontal and a parietal burr hole and in one case (0.4%) a mini-craniotomy was performed after initially starting with a single burr hole.

### Position of the subdural drain

In most cases (173/77.9%), the SDD crossed the central motor cortex along its intracranial course. In 192 cases (86.6%), the SDD was running in a straight line within the subdural space; in 30 cases (13.5%), the drain’s course appeared unfavourable and thus lead to focal compression of the brain cortex. In 24 cases (10.04%), the drain did not touch any cortical surface at the time of postoperative CT scan and in four cases (1.67%) it was lying within a cerebral sulcus.

The SDD’s intracranial entry angle varied between 0° and 103° (mean 39.39°) in axial and between 0° and 94° (mean 55.42°) in coronal planes. The angle between the drain and the cortex surface varied between 0° and 93° (mean 45.07°) in axial and between 0° and 99° (mean 54.36°) in coronal planes.

### Complications

Clinically relevant infections occurred in 27 patients (18.62%) during the postoperative course. Of these, twelve cases were urinary tract infections, eight patients developed pneumonia, one patient presented with meningitis and six patients showed a wound healing disorder of which two developed subdural empyema. Concerning CCT morphological characteristics small amounts of fresh blood within subdural space were seen in 80 cases (33.47%). Six postoperative CCT scans (2.51%) showed small intraparenchymal haemorrhages. In twelve patients (5.02%) relevant amounts of air were trapped intracranially, causing a space-occupying effect.

Due to recurrent or residual haematomas 38 patients (26.21%) had to undergo a second surgery. A significantly higher rate of haematoma recurrence or residuum was observed in the group of patients that were treated with a twist drill burr hole (12 of 22 cases, *p* < 0.001).

Nineteen patients (9.0%) experienced an epileptic seizure as postoperative complication. Sixteen of these patients (75.83%) received an anticonvulsant medication in consequence, in three patients no anticonvulsive therapy was initiated. In all cases, the SDD was removed immediately after the seizure. Two patients suffered from repetitive seizures, so that anticonvulsant therapy had to be escalated.

Using univariate logistic regression analysis, the following variables did not show a significant correlation with the occurrence of postoperative seizures (Table 1): age; gender; known epilepsy or intake of potentially epileptogenic medication; alcohol abuse; haematoma thickness and midline shift (pre- and postoperatively); haematoma density in CCT and localisation of the haematoma; one or two burr holes; twist-drill burr hole; localisation of the SDD; entering angle and tip-to-cortex angle of the SDD; amount of postoperative intracranial air; and serum levels of sodium, potassium, calcium, chloride and glucose.

**Table 1:** *P*-values of non-significant parameters for occurrence of postoperative seizures in regression analyses of a total of 211 patients (192 without, 19 with postoperative seizures). Regression models for haematoma-specific parameters were applied separately for left- and right-sided haematomas

General parameters	*P*-values in univariate regression	
Age		
< 30 (2)	0.232	
40–50 (4)	0.999	
50–60 (12)	1.000	
60–70 (35)	0.999	
70–80 (85)	0.999	
> 80 (73)	0.999	
Gender		
Male (145)	0.929	
Female (66)		
Pre-existent epilepsy		
Yes (12)	0.050	
No (199)		
Pre-existent alcoholism		
Yes (4)	0.341	
No (207)		
Epileptogenic medication		
Yes (28)	0.995	
No (183)		
Laterality of haematoma		
Unilateral left (98)	0.172	
Unilateral right (76)	0.094	
Bilateral (37)	0.714	
Biochemical markers		
Sodium	0.726	
Potassium	0.478	
Chloride	0.586	
Calcium	0.589	
Blood glucose	0.064	
Haematoma-specific parameters	*P*-values in univariate regression of left sided haematomas(*n* = 135)	*P*-values in univariate regression of right sided haematomas(*n* = 113)
Density in CCT (*n* = right/n = left)		
Isodense (8/6)	0.662	0.990
Hypodense (38/29)	0.999	0.999
Hyperdense (7/12)	0.999	1.000
Inhomogenic isodense (13/10)	0.999	1.000
Inhomogenic hypodense (69/56)	0.999	0.999
Haematoma thickness		
Axial	0.251	0.450
Coronal	0.346	0.798
SDD over eloquent areas		
Yes (91/82)	0.963	0.691
No (24/25)		
SDD insertion angle		
Axial	0.229	0.213
Coronal	0.134	0.668
SDD tip to cortex angle		
Axial	0.605	0.544
Coronal	0.107	0.129

Significance in univariate logistic regression analysis of risk factors for postoperative epileptic seizures was found in cases of craniotomy (vs. burr hole approach) (*p* = 0.037), especially in patients with craniotomy of left-sided haematomas (*p* = 0.03) and presence of subacute or acute subdural haematoma in the postoperative CCT scan (*p* = 0.021) (Table 2). No significance was found in univariate regression analyses of right-sided haematomas.

**Table 2:** *P*-values of risk factors for occurrence of postoperative seizures in regression analyses of a total of 211 patients (192 without, 19 with postoperative seizures). Regression models were applied separately for left- and right-sided haematomas as well as for the total of all haematomas. Statistically significant results are marked by *

	Univariate regression (left side)	Univariate regression (right side)	Multiple regression (left side)	Multiple regression (right side)
Unfavourable course of the SDD	*p* = 0.397	*p* = 0.999	*p* = 0.033*	*p* = 0.999
Craniotomy	*p* = 0.03*	*p* = 0.998	*p* = 0.016*	*p* = 0.998
	Univariate regression total		Multiple regression total	
Acute/subacute bleeding postop	*p* = 0.021*		*p* = 0.999	

To further investigate the above-mentioned results, multiple logistic regression analysis was applied after pairwise associations between potential covariates were assessed. In this context, subacute or acute bleeding in postoperative CCT scans and craniotomy were found to be associated strongly and therefore were not considered in multiple regression analysis to avoid multicollinearity.

A significantly higher risk for postoperative seizures was shown in the case of craniotomy of left-sided haematomas (*p* = 0.016) and when postoperative CCT scans showed an unfavourable course of the SDD on the left hemisphere (*p* = 0.033).

## Discussion

We report on a large series of consecutive patients who were surgically treated for cSDHs and investigated surgical and non-surgical risk factors for postoperative seizures. In our patient cohort, we found no elevated postoperative seizure rate in patients that were initially assumed to be at higher risk (alcoholics, epileptics, patients with electrolyte imbalances). In accordance with previously published studies, patients who were treated via an osteoplastic craniotomy had higher postoperative seizure rates. Furthermore, a higher seizure rate occurred in case of an unfavourable course of the SDD.

Our findings suggest, that postoperative seizures after the evacuation of chronic subdural haematoma are to a lesser extent determined by the patient’s individual risk factors but are rather influenced by surgical factors, which should therefore be considered carefully when treating these patients.

In our cohort of 211 consecutive patients, the incidence of postoperative seizures after evacuation of cSDHs was 9%. Compared to a relatively wide range of incidences in literature of 2.3 – 17%, our data is settled in a middle range [2, 6, 10]. The variety of surgical treatment options with different approaches and drain management may take account for the divergence of incidence. In consistency with other studies, our patients had a significantly increased seizure risk if an osteoplastic craniotomy was chosen as a surgical approach, especially on the left side [6, 11]. No significant correlation was found when analysing right-sided haematomas. An attempt at an explanation for this difference regarding haematoma laterality was given by Gobelny et al. who described similar findings in their retrospective study [12]. They assumed, that the postoperative seizure rate might be underestimated in patients with right-sided lesions, because of less evident clinical symptoms of seizures originating in the non-dominant hemisphere. Since the combination of craniotomy and insertion of a SDD into the subdural space correlated with a higher rate of fresh subdural bleedings, we assume that the latter causes a diminishing of the convulsive threshold.

Paying special attention to the intracranial course of the SDD we found an unfavourable course of the drain to be associated with a significantly higher rate of postoperative seizures, when the haematoma was situated on the left hemisphere. Possibly, misplacement and/or an irregular course of the SDD may result in focal compression of cortical tissue and thus trigger epileptic seizures. These findings emphasise the importance of a correct placement of the SDD when inserted into the subdural space and served the authors of this article as an argument for an alternative drain management of placing the drain into the subperiosteal layer as promoted by other groups [13–16].

Omitting the placement of a drain at all was shown to result in higher recurrence rates [17]. Consistently, we found a higher rate of haematoma recurrence in our cohort, if a twist drill burr hole without placing a drain was performed. However, relevant comorbidities and possibly compromised coagulation capacity in patients treated via twist drill burr hole may account for the higher recurrence rate of haematomas in these cases. Concerning postoperative seizures, we did not observe a higher rate in this subgroup.

The main limitation of this study is its retrospective design with a possible lack of information in our documentation on the occurrence of unnoticed and therefore untreated postoperative seizures. Most patients did not need continued monitoring of vital parameters during their postoperative course, so that episodes of short focal seizures might not have found their way in the patient’s record. This could be a reason for the differences of postoperative seizure rates regarding the laterality of haematomas, as mentioned above. The choice surgical approach could represent a selection bias, since the treating surgeons were more likely to choose minicraniotomy when acute bleeding components and septations were present within the haematoma, which may be associated with higher seizure rates per se.In addition, the assessment of the intracranial course of the SDD and its description as an unfavourable or meandered course is to some degree subjective, so that the generalizability of the respective findings in our study is limited.

## Conclusion

Although treating cSDHs is part of everyday practice in a neurosurgical unit, our findings underline the importance of an ongoing reflection of the routinely applied treatment modalities. With regard to surgically influenceable factors and postoperative seizure rates, osteoplastic craniotomy seems to be disadvantageous, particularly as multi-layered heterogenous haematomas—a situation when mini-craniotomy appears to be a feasible approach—were not associated with higher seizure rates. Additionally, we stress the importance of a cautious placement of SDDs since an unfavourable course of the drain over the left hemisphere was associated with a significant higher rate of postoperative seizures in our cohort. Against this background and similar findings in recent literature, placing the SDD into the subperiosteal layer after burr hole evacuation of a cSDH seems to be an appropriate alternative.

## Data Availability

All data and materials as well as software application support their published claims and comply with field standards.
